# MicroRNA signature of the human developing pancreas

**DOI:** 10.1186/1471-2164-11-509

**Published:** 2010-09-22

**Authors:** Samuel Rosero, Valia Bravo-Egana, Zhijie Jiang, Sawsan Khuri, Nicholas Tsinoremas, Dagmar Klein, Eduardo Sabates, Mayrin Correa-Medina, Camillo Ricordi, Juan Domínguez-Bendala, Juan Diez, Ricardo L Pastori

**Affiliations:** 1 Diabetes Research Institute, University of Miami, Miller School of Medicine, Miami FL, 33136, USA; 2 Center for Computational Science, University of Miami, Miami FL, 33136, USA; 3 Dr. John T. Macdonald Foundation Department of Human Genetics, University of Miami, Miller School of Medicine, Miami FL, 33136, USA

## Abstract

**Background:**

MicroRNAs are non-coding RNAs that regulate gene expression including differentiation and development by either inhibiting translation or inducing target degradation. The aim of this study is to determine the microRNA expression signature during human pancreatic development and to identify potential microRNA gene targets calculating correlations between the signature microRNAs and their corresponding mRNA targets, predicted by bioinformatics, in genome-wide RNA microarray study.

**Results:**

The microRNA signature of human fetal pancreatic samples 10-22 weeks of gestational age (wga), was obtained by PCR-based high throughput screening with Taqman Low Density Arrays. This method led to identification of 212 microRNAs. The microRNAs were classified in 3 groups: Group number I contains 4 microRNAs with the increasing profile; II, 35 microRNAs with decreasing profile and III with 173 microRNAs, which remain unchanged. We calculated Pearson correlations between the expression profile of microRNAs and target mRNAs, predicted by TargetScan 5.1 and miRBase altgorithms, using genome-wide mRNA expression data. Group I correlated with the decreasing expression of 142 target mRNAs and Group II with the increasing expression of 876 target mRNAs. Most microRNAs correlate with multiple targets, just as mRNAs are targeted by multiple microRNAs. Among the identified targets are the genes and transcription factors known to play an essential role in pancreatic development.

**Conclusions:**

We have determined specific groups of microRNAs in human fetal pancreas that change the degree of their expression throughout the development. A negative correlative analysis suggests an intertwined network of microRNAs and mRNAs collaborating with each other. This study provides information leading to potential two-way level of combinatorial control regulating gene expression through microRNAs targeting multiple mRNAs and, conversely, target mRNAs regulated in parallel by other microRNAs as well. This study may further the understanding of gene expression regulation in the human developing pancreas.

## Background

MicroRNAs are small non-coding RNAs [1-3] that act mostly as translational repressors through binding to partially complementary sequences of target messenger RNAs. Recent studies indicate that in some cases microRNAs might act also as positive regulators of translation and transcription [4,5]. To date, more than 700 human microRNAs are annotated in the Wellcome Trust Sanger Institute microRNA database [6]. MicroRNAs play a fundamental role in regulation of gene expression, consequently affecting key biological events such as embryogenesis, stem cell proliferation/differentiation and organ development [7]. The method of conditional knockout of the microRNA-processing enzyme Dicer has been used to study the action of microRNAs in specific tissues and organs. This approach has confirmed that the expression of microRNAs is essential for morphogenesis of several systems, including the lungs, limbs, muscle, skin and neuronal development and differentiation [8-12] including the genesis of pancreatic islet cells [13]. Dicer-null animals displayed gross defects in all pancreatic lineages; the most dramatic reduction was seen in the endocrine cells, especially the insulin-producing beta cells. Another approach utilized to assess the role of microRNAs in organs/tissues is the loss of function, i.e., silencing or knock out of a single or cluster microRNAs. For instance, genetic deletion of the miR-1/miR-133 cluster, showed its crucial role in cardiac skeletal muscle development [14]. Several microRNAs have been described in the context of pancreatic development, pancreatic exocrine/endocrine microRNA expression or islet biology/physiology. For example, knockdown of miR-375, an islet microRNA that negatively controls insulin secretion [15], has a deleterious effect on the developing pancreas of zebra fish [16]. Mice lacking miR-375 (375KO) are hyperglycemic and exhibit an increase in total pancreatic alpha-cell numbers, whereas the pancreatic beta-cell mass is decreased [17]. Recent studies indicate that during human pancreatic development miR-7, miR-9, miR-375 and miR-376 are specific islet microRNAs expressed at high levels [18,19]. MiR-124a is known to target FOXA2 the transcription factor crucial for early pancreatic formation [20] by affecting genes such as PDX1, KCNJ11 (Kir6.2) and SUR1, essential for the normal development of the pancreas, glucose metabolism and insulin secretion [21], while miR-15a, -15b, -16 and -195 have important roles in regulating translation of NGN3, a transcription factor affecting the adoption of an endocrine fate during embryogenesis [22].

Research conducted over the last decade has outlined rather comprehensive "roadmap" of the major molecular events that shape mouse islet development [23,24]. Development of the human endocrine pancreas has not been studied as well as that of the mouse. The previous work has described expression of endocrine hormones as well as transcription factors, and presented morphological examination of islet formation during human fetal pancreatic development [25-29].

In this study we investigated the global expression profile of microRNAs in the human fetal pancreas from age 10 to 22 weeks of gestational age (wga) and found 212 microRNAs expressed throughout this entire period. Understanding the complex processes behind pancreatic development will require the identification of RNA targets that are controlled by the extensive microRNA network expressed throughout the process. To identify potential microRNA gene targets, we calculated the correlations between the expression profile of these 212 microRNAs and their corresponding mRNA targets, predicted by bioinformatic tools, that were found in the genome-wide RNA microarray study by Sarkar et al. [28]. These studies will advance our understanding of pancreatic development, potentially unveiling prospective therapeutic targets to treat diabetes.

## Results and discussion

### MicroRNA Expression profile during pancreatic development

Using TaqMan Low Density Arrays (TLDA) from Applied Biosystems we have performed microRNA arrays with 10 samples acquired at different stages of human fetal pancreatic development. Specifically; 10 wga (two samples), 11 wga (one sample), 13 wga(one sample), 14 wga (three samples), 15 wga (one sample), 21 wga (one sample), 22 wga (one sample). The TLDA platform has been selected because it requires smaller amounts of RNA ~100 ng per experiment, which is about ~20 times less than what is required for other microarray hybridization platforms, furthermore it delivers quantitative output. We identified 212 microRNAs expressed throughout all studied gestational ages. Interestingly several microRNAs reported previously in studies of mouse pancreatic development/regeneration and islet function were also found expressed during human pancreatic development (Table 1).

**Table 1:** MicroRNAs expressed during human fetal pancreatic development.

miR-7	Expressed in pancreatic adult and fetal endocrine cells [18,19,30]
miR-375	Negative regulator of glucose-induced insulin secretion through myothrophin regulation [15]. miR-375 K/O mice are hyperglycemic -more alpha cells; less beta-cells- [17]. Regulation of PI3 pathway by regulation of PDK1 in insulinoma cells [31]. The miR-375 gene promoter directs expression selectively to endocrine pancreas [32].
miR-9	Expressed at high levels during islet development [19]. Target of transcription factor Onecut-2 impairing glucose-induced insulin secretion in insulinoma cells.
miR-195; miR-16 miR-15a; miR-15b	Role in pancreatic regeneration, possibly by targeting Ngn3 [22].
miR-124a	Regulation of insulin secretion machinery and transcription factor Foxa2 in insulinoma cells [21,33].
miR-218	Expressed in mouse early fetal pancreas, controls the liver and pancreatic development regulator Onecut-2 in liver embryonic cells [34].
miR-484; miR-107; miR-30d	High glucose down-regulates their expression in insulinoma cells [35].
miR-146a	Increased expression in islets from db/db obese mice, contributes to fatty acids-induced beta-cell dysfunction [36]. Pro-inflammatory cytokines induce its expression in human islet and MIN6 cells [37].
miR-29a	Over-expression induced insulin resistance in 3T3 adipocytes [38].
miR-503	miR-503 is expressed in a pattern similar to that of miR-375 in a mouse progenitor cells at e14.5 pancreas [13].
miR-376a	Expressed at high levels during islet development [19].
miR-21; miR-34a	Pro-inflammatory cytokines induce its expression in human islet and MIN6 cells [37]. miR-34a also contributes to fatty acids-induced beta-cell dysfunction [36].
miR-96	Increases mRNA and protein levels of granulophilin, a negative regulator of insulin exocytosis [33].

These microRNAs have been previously described as expressed in adult or developing pancreatic tissue and/or having a functional role in islets/insulinoma cells.

For the correlation analysis we arranged the samples in 3 gestational periods: 10-11wga (three samples); 13-15 wga (five samples) and 21-22 wga (two samples) and classified the microRNAs in three groups according to their expression profile (Material and Methods). Groups I and II contain 4 and 35 microRNAs respectively. Their statistically significant (p < 0.05) expression change was either up-regulated (group I) or down-regulated (group II) at least from one gestational stage to the other. Group III comprises of 173 microRNAs with expression relatively unchanged (p > 0.05) (Table 2 & Additional file 1**, Table S1)**. Table 2 was prepared using relative quantification (RQ) values normalized by nucleolar RNA (RNU48) as endogenous control (Additional file 1**, Table S1**). RQ is a measure of the abundance of microRNA transcripts at each developmental stage. The microRNAs in Table 2 are arranged according to their descending RQ starting at the stage 10 wga. MiR-7, miR-124, miR-9 and miR-375, are microRNAs previously reported as related to pancreatic islets [18-20]. We found that only statistically significant increase of expression was for miR-7. miR-375 showed an increasing trend, however slightly below the threshold value (p = 0.08), which might be the result of a low sample number. Additional file 2**, Table S2 **shows the average RQs for microRNAs in group I and II in each gestational period.

**Table 2:** MicroRNA groups classified according to their expression profiles.

Group I (4)	Group II (35)	Group III (173)				
miR-7	miR-92	miR-26a	miR-410	let-7g	miR-142-5p	miR-326
miR-141	miR-214	miR-19b	miR-200b	miR-148b	miR-615	miR-488
miR-98	miR-484	miR-126	miR-433	let-7a	miR-486	miR-629
miR-489	miR-125a	miR-125b	miR-142-3p	miR-532	miR-143	miR-369-3p
	miR-335	miR-30c	miR-186	miR-379	miR-656	miR-496
	miR-218	miR-16	miR-140	miR-565	miR-95	miR-133a
	miR-99b	miR-24	miR-152	miR-20b	miR-490	miR-203
	miR-342	miR-200c	miR-411	miR-32	miR-101	miR-378
	miR-382	miR-199a	miR-365	miR-424	miR-107	miR-511
	miR-301	miR-100	miR-374	miR-425-5p	miR-193a	miR-520g
	miR-432	miR-99a	miR-324-3p	miR-361	miR-383	miR-17-3p
	miR-181b	miR-26b	miR-27b	miR-572	miR-193b	miR-190
	miR-323	miR-594	miR-10a	miR-30e-5p	miR-339	miR-450
	miR-103	miR-127	miR-320	miR-423	let-7e	miR-518b
	miR-21	miR-20a	miR-134	miR-10b	let-7d	miR-659
	miR-130b	miR-130a	miR-429	miR-183	miR-372	miR-376b
	miR-135b	miR-30b	miR-137	miR-146a	miR-213	miR-338
	miR-182	miR-30a-5p	miR-149	miR-132	miR-491	miR-504
	miR-181d	miR-148a	miR-324-5p	miR-195	miR-503	miR-576
	miR-25	miR-485-3p	miR-145	miR-451	miR-224	miR-545
	miR-17-5p	miR-30d	miR-151	miR-485-5p	miR-500	miR-219
	miR-296	miR-200a	miR-210	miR-199b	miR-493	miR-518e
	miR-135a	miR-19a	miR-204	miR-23a	miR-215	miR-642
	miR-345	miR-223	miR-660	miR-194	miR-425	miR-196b
	miR-299-5p	miR-106b	miR-328	miR-15a	miR-1	miR-422a
	miR-18a	miR-331	miR-146b	miR-181c	miR-502	miR-518c
	miR-369-5p	miR-375	miR-192	let-7f	miR-206	miR-337
	miR-519d	miR-197	miR-221	miR-340	miR-650	miR-189
	miR-494	miR-191	let-7c	miR-362	miR-29c	miR-509
	miR-550	miR-93	miR-30e-3p	miR-22	miR-124a	miR-299-3p
	miR-187	miR-27a	miR-9	miR-654	miR-452	miR-381
	miR-409-5p	miR-376a	miR-30a-3p	miR-330	miR-133b	miR-329
	miR-380-5p	miR-487b	miR-31	miR-155	miR-139	miR-518f
	miR-512-3p	miR-15b	miR-28	miR-501	miR-29a	
	miR-96	miR-222	miR-23b	hsa-let-7b	miR-542-5p	

MicroRNAs from group I and II increase and decrease their expressions respectively throughout the entire studied period. Group III includes microRNAs with relatively constant expression throughout the period studied.

**Figure 1:**
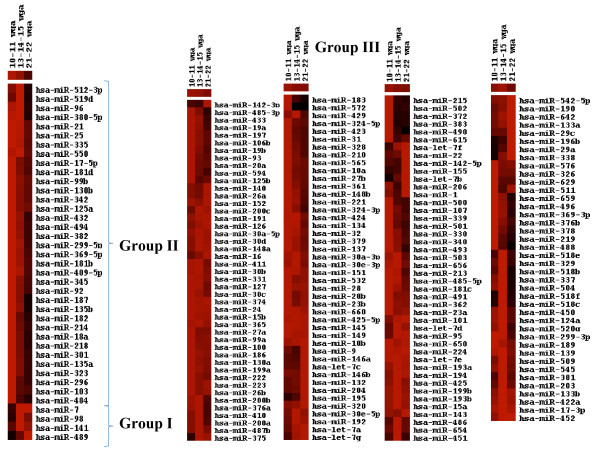
**Expression of microRNAs during human pancreatic development**. The Heat- map figures were obtained using Cluster and Treeview software [44] with the data presented in Additional file 1, Table S1. This table shows correlation among expression profiles of microRNAs comprised into three groups from all studied fetal pancreatic samples; Group I, Group II and Group III. The color of each cell represents the expression of microRNA normalized to small nucleolar RNU48 RNA. Colorgram depicts intensity of expression from high (bright red) to low (black). Please note that even though in some cases the group III colorgram suggests a change of expression throughout the gestational stages (e.g miR-376, miR-378, etc), these were found to be not statistically significant.

A hierarchical cluster representation of microRNAs clearly shows the pattern of expression of these three groups (Figure. 1).

### Inverse correlation between microRNA and mRNA during human pancreatic development

Although mechanistically, microRNAs inhibit mRNA translation, the expression of a given microRNA has also been associated with the downregulation of its target mRNA levels [30,31]. Therefore, to identify potential microRNA targets, we performed a statistical analysis looking for inverse correlations between the microRNAs in groups I and II (a total of 39 microRNAs) and the algorithm-predicted target genes that were found in a human pancreatic development genome-wide mRNA microarray gene expression study [28]. Predictive algorithms are a powerful tool to identify potential RNA targets [32]. The target RNAs were selected by two of the most commonly used computational microRNA predictive target programs, miRBase [33] and TargetScan [30,34]. There is a limitation to this kind of analysis because it utilizes only the sequence correlation of 3'UTR mRNA domain with microRNA seed sequences. This excludes naturally occurring microRNA targets with recognition motifs within the gene coding sequence. This kind of microRNA/mRNA interaction has been recently reported as well [35]. Computational predictions often generate long lists of target genes and these are not always concordant [32]. Therefore, the correlations were calculated with target genes predicted by each program individually, and also by the intersection of both algorithms, as this might increase the odds of identifying the *bona fide *RNA targets [36].

High confidence correlation (R^2^≥0.8) analysis between microRNAs and gene targets identified 2832 and 9448 correlations using miRBase and TargetScan respectively (Additional file 3, **Table S3 and **Additional file 4**, Table S4)**. A total of 1018 genes were identified as potential targets by both predictive algorithms (Additional file 5**, Table S5**); microRNAs from group I correlated with decreased expression of 142 potential targets while micoRNAs from group II, correlated with the increasing expression of 876 potential targets (Table 3). For all microRNA groups the number of targets predicted by TargetScan was higher than by miRBase, which is in agreement with previous observations [37,38].

**Table 3:** Correlation between microRNAs and gene targets.

	Genes Group I				Genes Group II		
miRNA	TargetScan	Sanger	Shared	miRNA	TargetScan	Sanger	Shared
miR-141	318	99	53	miR-17-5p	433	121	65
miR-7	463	152	43	miR-181d	349	114	55
miR-489	209	95	34	miR-296	382	97	53
miR-98	105	55	12	miR-214	513	92	48
				miR-103	384	115	46
				miR-181b	284	88	46
				miR-125a	385	100	45
				miR-484	455	82	40
				miR-345	263	71	34
				miR-301	215	69	28
				miR-182	291	77	27
				miR-18a	231	98	27
				miR-218	248	89	27
				miR-342	417	72	24
				miR-130b	221	92	23
				miR-135a	231	74	23
				miR-323	274	67	22
				miR-494	385	47	22
				miR-382	193	58	21
				miR-432	241	60	21
				miR-135b	190	59	19
				miR-25	151	46	17
				miR-92	200	67	17
				miR-21	106	56	15
				miR-299-5p	162	64	15
				miR-335	220	45	14
				miR-187	60	71	12
				miR-512-3p	120	19	12
				miR-519d	139	28	11
				miR-550	150	31	11
				miR-96	124	47	11
				miR-409-5p	85	55	10
				miR-99b	32	76	7
				miR-380-5p	200	30	6
				miR-369-5p	19	54	2
Total	1095	401	142	Total	8353	2431	876

Data for this table was obtained setting inverse correlations with R^2 ^> = 0.80 as a cut off high confidence level.

### Genes involved in pancreatic development correlated with microRNAs

**Figure 2:**
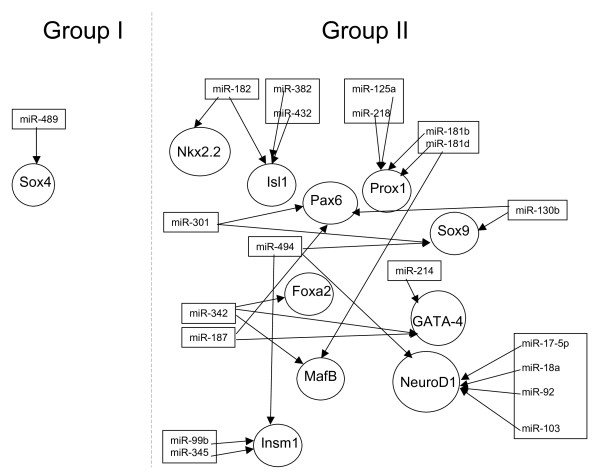
**Network of microRNA and mRNA targets**. MicroRNAs (group I &II) and their predicted mRNA targets collaborate with each other during pancreatic development.

We searched for microRNAs that negatively correlated with genes from signaling pathways and transcription factors that are known to be involved in murine pancreatic development and/or regulate beta-cell development [39] (Table 4). Out of the 28 candidate genes, 11 were identified as potential targets for microRNAs. Most of the correlations corresponded to microRNAs from group II. Ten genes up-regulated throughout the development correlated with 19 decreasing microRNAs from group II. Decreasing gene SOX4, was identified as a potential target for miR-489 from group I. The identification of 19 microRNAs with decreasing expression along the pancreatic development, thus contributing to the upregulation of their potential targets, suggests a collaborative network of microRNAs and mRNAs during this period (Figure. 2). Most microRNAs target more than one gene and conversely a given mRNA could be the target of several microRNAs. For example, NEUROD1, a key transcription factor involved in the conversion of pancreatic progenitor cells into endocrine cells [40] was identified as a potential target of five microRNAs: miR-17-5p, miR-18a, miR-92, miR-103 and miR-494. The decrease in the expression of these five microRNAs would lead to the up-regulation of NEUROD1. It is apparent that microRNAs can target genes/transcription factors that have a role at different stages of embryogenesis, for example miR-342 targets genes such as GATA-4, involved in the formation of definitive endoderm [41] and transcription factors FOXA2 and MAFB both involved in beta-cell differentiation and maturation [39,42].

**Table 4:** Genes involved in pancreatic development correlated with microRNAs.

Transcription factors	Sanger	TargetScan
Isl1	miR-182 (II)	miR-382 (II), miR-432 (II)
Hlxb9	0	0
Hex	0	0
Prox1	miR-181b (II), miR-181 d (II)	miR-125a (II), miR-218 (II)
Hnf1β	0	0
Hnf6	0	0
Ptf1a	0	0
Pdx1	0	0
Pbx1	0	0
Sox9	0	miR-130b (II), miR-301 (II), miR-494 (II)
Sox4	0	miR-489 (I)
GATA-4	0	miR-187 (II), miR-214 (II), miR-342 (II)
GATA-6	0	0
Ngn3	0	0
NeuroD1	0	miR-17-5p (II), miR-18a (II), miR-92 (II), miR-103 (II), miR-494 (II)
Insm1	miR-99b (II)	miR-99b (II), miR-345 (II), miR-494 (II)
Myt1	0	0
Pax6	miR-187 (II)	miR-130b (II), miR-301 (II)
Pax4	0	0
Nkx2.2	miR-182 (II)	0
Nkx6.1	0	0
MafA	0	0
MafB	0	miR-181 d (II), miR-342 (II)
Foxa1	0	0
Foxa2	0	miR-342 (II)
HNF1α	0	0
HNF4α	0	0
TCF7L2	0	0

Data for this table was obtained setting inverse correlations with R^2 ^> = 0.8 as a cut off high confidence level. Numbers in parenthesis indicates the microRNA group.

## Conclusions

In this study we determined the microRNA expression signature during human pancreatic development, identifying a total of 212 microRNAs expressed at least once in one of the four stages evaluated (10 to 22 wga). To further our understanding of gene expression regulation in the human developing pancreas, we compared this microRNA signature with the global expression of genes during the similar period of the developing pancreas. There are potential limitations to this kind of study that should be considered: 1) the study was designed under the assumption that microRNAs are associated with downregulation of their targets. However, some microRNAs were reported as positive regulators of transcription or translation [4,5], 2) the correlation between microRNAs and gene expression was performed with mRNAs only, but there are some microRNAs that regulate only protein translation; 3) all potential targets were identified by algorithms analyzing the 3'UTR of the mRNA, while some microRNAs can also interact with targets containing the recognition sequence in the coding region; 4) the expression of microRNAs and mRNAs was evaluated on different set of sample tissues, using different platforms by different laboratories; 5) in this study we have analyzed the pancreas as a whole, instead of looking at the cellular subsets. Therefore, it is not possible to determine if the observed changes corresponded to a specific cell population (e.g endocrine progenitors, mesenchymal tissue etc).

Despite of these caveats, the correlation between the expression of hundreds of microRNAs with the analysis of thousands of genes expressed during human pancreatic development suggests the intertwined collaborative network of microRNAs and mRNAs. It also provides broad and useful information to explore potential two-way level of gene expression control regulation in which microRNAs target multiple mRNAs, and, target mRNAs are regulated in parallel by multiple microRNAs. Ultimately, the speculation regarding the roles of all these presumptive targets will be justified only if they are experimentally validated.

## Methods

### Human fetal pancreas procurement

Human fetal pancreases from 10 to 22 weeks of gestational age (wga) were collected from fetal tissue immediately after elective termination of pregnancy. The healthy women admitted to local clinics were properly informed and gave their consent to use fetal tissues for research studies. The study is in compliance with US legislation and the guidelines of the University of Miami. Gestational age was determined on the basis of the last menstrual period, with ultrasonographic measurements of the Crown-Rump Length, and the biparietal diameter.

### MicroRNA isolation

MicroRNA isolation was performed using mirVana microRNA Isolation kit from Ambion/Applied Biosystems (Foster City CA), following protocol for the total RNA isolation procedure provided by the manufacturer.

### Quantitative microRNA profiling: criteria for inclusion of microRNAs in this study

We performed this study with 10 human fetal pancreases of following gestational stages: two samples (10 wga); one sample (11 wga); one sample (13 wga); three samples (14 wga); one sample (15 wga); one sample (21 wga) and one sample (22 wga). Total RNA was isolated using mirVana microRNA Isolation kit, cDNA synthesis and the PCR amplification was performed according to the manufacturer's instructions (Applied Biosystems, Foster City, CA). MicroRNA profiling was performed with micro fluidic cards TaqMan^® ^Low Density Array (TLDA, v1.0) for human microRNAs, which allows quantitative assessment of 352 microRNAs using the AB7900 instrument (Applied Biosystems). Quantitative values were calculated as relative quantification (RQ), normalized to endogenous control small nucleolar RNA RNU48, which is expressed evenly in all samples. RQs were calculated with SDS software supplied by the manufacturer (Applied Biosystems), utilizing the equation RQ = 2^-ΔCt ^[43], where Ct is the number of cycles at which the sample reaches a software-determined threshold within the exponential amplification phase. Ct ≥ 35 cycles was considered as undetermined. The Gene Expression Omnibus (GEO) accession number is GSE22026.

For the correlation analysis we arranged the samples in 3 gestational periods: 10-11 wga; 13-15 wga and 21-22 wga. Only microRNAs that amplified in 2 out 3 samples for 10-11 wga period, 4 out of 5 samples for 13-15 wga and 2 out of 2 samples in 21-22 wga were considered as positively expressed. Statistical analysis between the groups: 10-11 wga vs 13-15 wga; 10-11 wga vs 21-22 wga and 13-15 wga vs 21-22 wga was performed with two-tailed Student's t test (Additional file 1**, Table S1**). Values were considered significant when p < 0.05. The microRNAs were classified according to their expression profile into three groups. Group I contained microRNAs with the expression increased at least from one gestational stage to the other (p < 0.05). Group II contained microRNAs with the expression decreased at least from one gestational stage to the other (p < 0.05). In group III we included the microRNAs with the expression unchanged throughout the studied gestational stages (p > 0.05).

### MicroRNA target gene databases

Two microRNA target databases were used in this analysis. One arch.v5.txt.homo_sapiens.zip, downloaded from Sanger miRBase Targets http://microrna.sanger.ac.uk/cgi-bin/targets/v5/download.pl; the other Nonconserved_Site_Context_Scores.zip, downloaded from TargetScan http://www.targetscan.org/cgi-bin/targetscan/data_download.cgi?db=vert_50.

### Correlation Analysis

mRNA gene expression files were retrieved from Sarkar SA, Kobberup S, Wong R, Lopez AD, Quayum N, Still T, Kutchma A, Jensen JN, Gianani R, Beattie GM *et al*: **Global gene expression profiling and histochemical analysis of the developing human fetal pancreas **[28]. 22,000 genes were measured by 54,675 probe-sets on Human Genome HG U133 Plus 2.0 microarrays (Affymetrix, Santa Clara, CA, USA). The mRNA hybridization data from genes with multiple probes were averaged prior to the correlation analysis. For the correlation analysis the Sarkar et al gene expression data were grouped in three periods: 9, 10 and 11 wga (9-11); 15 wga; and 20, 23 wga. The gene expression of these three periods was compared to the microRNA expression corresponding to the three following periods: 10-11 wga; 13-15 wga and 21-22 wga. The Pearson correlation of the expression profile between microRNA and a target gene was calculated in the R package. The correlation coefficient (r) was calculated to determine the regulatory role of the microRNA on its target genes. The correlation with r close to -1 depicts a strong negative correlation between microRNA and its target gene, indicating the suppression function of microRNA on its target gene. *P*-value is used to test H^0^: r = 0. Benjamini-Hochberg FDR was used to correct the original p-value for multiple test correction. The correlations with adjusted *p*-value < 0.05 are significant correlations, and among these correlations the ones with (R^2^≥0.8) are significantly negative correlations where microRNAs suppress the expression of target genes in terms of expression in the three gestational ages.

## Abbreviations of genes referenced in this study

Isl1: Insulin gene enhancer protein; Hlxb9: homeo box HB9; Hex (Hhex):hematopoietically expressed homeobox protein; Prox1: Homeobox prospero-like protein; HNF1β: hepatocyte nuclear factor 1 homeobox B; HNF6: hepatocyte nuclear factor 6; Ptf1a: Pancreas specific transcription factor, 1a; Pdx1: Pancreatic and duodenal homeobox 1; Pbx1: Pre-B-cell leukemia transcription factor 1; Sox9: transcription factor sox9; Sox4:transcription factor sox4; GATA-4: transcription factor gata-4; GATA-6: transcription factor gata-6; Ngn3: neurogenin-3; NeuroD1: Neurogenic differentiation 1; Insm1: Insulinoma-associated protein 1; Myt1: Myelin transcription factor 1; Pax6: Paired box gene 6: Pax4: Paired box gene 4; Nkx2.2: Homeobox protein Nkx-2.2; Nkx6.1: Homeobox protein Nkx-6.1; MafA: V-maf musculoaponeurotic fibrosarcoma oncogene homolog a (avian); MafB: V-maf musculoaponeurotic fibrosarcoma oncogene homolog b (avian); Foxa1: forkhead box A1; Foxa2: forkhead box A2; HNF1α: hepatocyte nuclear factor 1 homeobox A; HNF4α: Hepatocyte nuclear factor 4 alpha; TCF7L2:Transcription factor 7-like 2 (T-cell specific, HMG-box); kir6.2: ATP-sensitive K+ channel; sur1: sulfonylurea receptor 1

## Competing interests

The authors declare that they have no competing interests.

## Authors' contributions

VBE and SR performed research, analyzed data and wrote the manuscript. ZJ, SK and NT performed the computational analysis. ES contributed to the correlation statistical analysis. DK analyzed data and wrote the manuscript. MCM collected the samples. CR analyzed the data. JDB analyzed the data and wrote the manuscript. JD supervised the collection of samples, analyzed the data and reviewed the manuscript. RLP initiated and supervised the research, analyzed the data and wrote the manuscript. All the authors read and approved the final version of the manuscript.

## Supplementary Material

Additional file 1 **Table S1: MicroRNA PCR array data of human developing pancreas**. PCR amplification cycles (CTs) were used to calculate RQs using small nucleolar RNU48 RNA as endogenous control. The t test between the different stages was calculated with individual RQs. Click here for file

Additional file 2 **Table S2: Average RQ values for microRNAs included in group I & II**. Click here for file

Additional file 3 **Table S3: Correlation between microRNAs and gene targets predicted by miRBase algorithm**. Click here for file

Additional file 4 **Table S4: Correlation between microRNAs and gene targets predicted by TargetScan algorithm**. Click here for file

Additional file 5 **Table S5: Correlation between microRNAs and gene targets predicted by both algorithms, miRBase and TargetScan**. In additional files 3, 4 and 5, the data for microRNAs are average RQs. Data for genes are hybridization intensity values from normalized arrays [28]. Click here for file
